# One-pot hydrothermal synthesis of CuBi co-doped mesoporous zeolite Beta for the removal of NO_x_ by selective catalytic reduction with ammonia

**DOI:** 10.1038/srep30132

**Published:** 2016-07-22

**Authors:** Zhiguo Xie, Xiaoxia Zhou, Huixia Wu, Lisong Chen, Han Zhao, Yan Liu, Linyu Pan, Hangrong Chen

**Affiliations:** 1 State Key Laboratory of High Performance Ceramics and Superfine Microstructure, Shanghai Institute of Ceramics, Chinese Academy of Sciences, 1295 Dingxi Road, Changning District, Shanghai 200050, P.R. China; 2 The Key Laboratory of Resource Chemistry of Ministry of Education and the Shanghai Key Laboratory of the Rare Earth Functional Materials. Shanghai Normal University, 100 Guilin Road, Xuhui District, Shanghai 200234, P.R. China; 3 National Synergetic Innovation Center for Advanced Materials (SICAM), Jiangsu, China

## Abstract

A series of CuBi co-doped mesoporous zeolite Beta (Cu_x_Bi_y_-mBeta) were prepared by a facile one-pot hydrothermal treatment approach and were characterized by XRD, N_2_ adsorption-desorption, TEM/SEM, XPS, H_2_-TPR, NH_3_-TPD and *in situ* DRIFTS. The catalysts Cu_x_Bi_y_-mBeta were applied to the removal of NO_x_ by selective catalytic reduction with ammonia (NH_3_-SCR), especially the optimized Cu_1_Bi_1_-mBeta achieved the high efficiency for the removal of NO_x_ and N_2_ selectivity, superior water and sulfur resistance as well as good durability. The excellent catalytic performance could be attributed to the acid sites of the support and the synergistic effect between copper and bismuth species. Moreover, *in situ* DRIFTS results showed that amides NH_2_ and NH_4_^+^ generated from NH_3_ adsorption could be responsible for the high selective catalytic reduction of NO_x_ to N_2_. In addition, a possible catalytic reaction mechanism on Cu_1_Bi_1_-mBeta for the removal of NO_x_ by NH_3_-SCR was proposed for explaining this catalytic process.

Nowadays, it is still of great challenges for the effectively catalytic purification of diesel exhausts, especially for the NO_x_ from diesel engine, since the conventional three-way catalysts are no longer effective in selective reducing NO_x_^1^. The commercial selective catalytic reduction with ammonia (NH_3_-SCR) catalyst for the removal of NO_x_, *i.e.*,V_2_O_5_-WO_3_/TiO_2_, only shows high catalytic efficiency in a narrow temperature window of 300–400 °C^2^, besides, the poor water and sulfur resistance as well as the toxicity of V_2_O_5_ also greatly prohibit the popularity of vanadium-based composite oxides. Therefore, researchers have devoted to develop a new kind of non-vanadia catalysts to overcome the disadvantages of vanadium-based composite oxides^3^. It was reported that compared to the traditional V_2_O_5_-WO_3_/TiO_2_, non-vanadia catalyst not only presents the wider NH_3_-SCR temperature windows with high N_2_ selectivity, but also owns good durability and strong resistance against H_2_O and SO_2_.

Very recently, zeolite-based catalysts with high surface areas and pore volume, abundant acidity sites, outstanding thermal and hydrothermal stability, as good catalyst supports, have attracted much research attention in selective catalytic reduction NO_x_ by ammonia^4^,^5^. However, small microporous channels of zeolite greatly prevented the diffusion and transport of some large molecules, resulting in the low catalytic performance in a great majority of traditional catalytic reactions^6^. Therefore, a novel zeolite with mesoporous structure has been developed, which combines the advantages of conventional crystalline zeolite and mesoporus material, to enable the quick access for the diffusion and transport attributed to the hierarchically porous structure. It is generally believed that mesoporous zeolites possess remarkably higher catalytic activity and longer catalytic lifetime than conventional zeolites owing to the crystalline framework and the hierarchically porous structure^7^. Thereinto, the mesoporous Beta zeolite (mBeta) with unique three-dimensional network of large pores (12MR) exhibits much high surface area and excellent hydrothermal stability, which is widely applied in fine chemistry^8^. In the past decades, a number of synthetic approaches of mBeta have been explored and some encouraging results have been obtained^8^,^9^,^10^. Such as, Xiao *et al*., synthesized highly mesoporous single-crystalline zeolite beta by using a commercial polymer, polydiallyldimethylammonium chloride (PDADMA) as both structure-directing agent and porogen, which showed better hydrothermal stability and higher catalytic activity than conventional zeolite Beta in large molecules involved acid-catalyzed reactions^8^. In addition, many metal oxides were reported to have good performance in the removal of NO_x_ by NH_3_-SCR, *e.g.*, Cu-loaded zeolite beta exhibited a good activity and hydrothermal stability in the NH_3_-SCR of NO_x_^11^,^12^. It is also reported that the introduction of Bi_2_O_3_ can improve the SO_2_ resistance^13^.

On the basis of our previous work^14^, a novel CuBi co-doped mesoporous zeolite Beta (Cu_x_Bi_y_-mBeta) has been synthesized by one-pot hydrothermal treatment approach, by which the copper and bismuth species can be well dispersed into the framework of mesoporous zeolite Beta. Therein, the optimized prepared catalyst Cu_1_Bi_1_-mBeta exhibits very high catalytic activity for the selective catalytic reduction of NO_x_ with NH_3_. In addition, the N_2_ selectivity, water vapor and sulfur resistance and durability of the Cu_1_Bi_1_-mBeta catalyst have been detailedly investigated. Finally, a possible catalytic mechanism of SCR of NO_x_ with ammonia on this prepared catalyst CuBi-mBeta is proposed to clarify the catalytic process.

## Results

### Structure Characteristics

**Figure 1:**
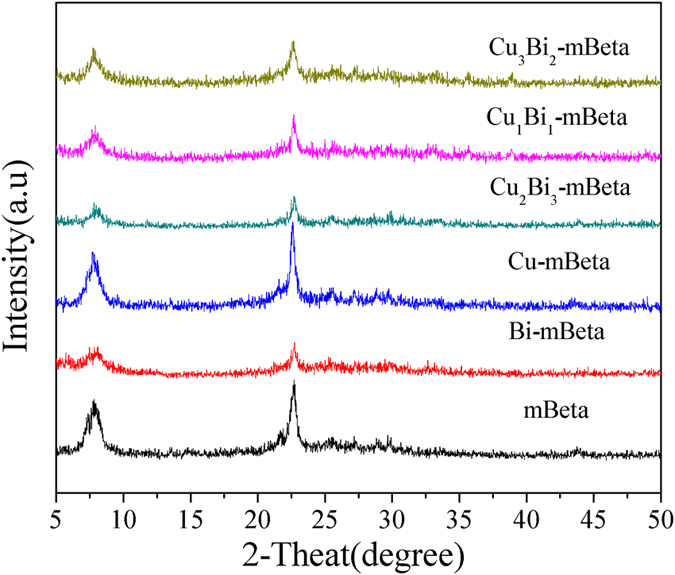
XRD patterns of samples mBeta, Cu-mBeta, Bi-mBeta and Cu_x_Bi_y_-mBeta.

The powder XRD patterns of mBeta, Cu-mBeta, Bi-mBeta and Cu_x_Bi_y_-mBeta are shown in Fig. 1. It is found that all prepared samples keep the diffraction peak of typical zeolite beta structure, and no diffraction peaks corresponding to copper and bismuth species can be detected. Therefore, it is believed that the copper and bismuth species could be well-incorporated into the framework of zeolite as ions or highly dispersed into the mesoporous channels as metal oxides. It is noted that compared with the mBeta, the doping of Bi species can cause the inevitable destruction of zeolite framework to a certain extent, and the intensity of XRD peak of Cu_x_Bi_y_-mBeta decreases with the increase of Bi-loading content, as shown in Fig. 1.

**Figure 2:**
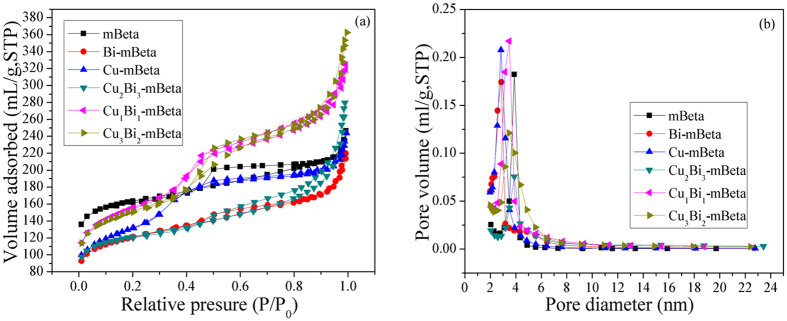
(**a**) N_2_ adsorption/desorption isotherms and (**b**) the corresponding pore size distributions of the samples.

The N_2_ adsorption isotherms and pore size distribution curves for samples mBeta, Cu-mBeta, Bi-mBeta and Cu_x_Bi_y_-mBeta are shown in Fig. 2 and the corresponding pore structure parameters of all the samples are summarized in Table 1. All the samples exhibit typical type IV isotherms, confirming the presence of mesoporous structure. The reference sample mBeta shows well-defined mesopore of 3.8 nm, and the BET surface area and total pore volume are calculated to be 556 m^2^/g and 0.34 cm^3^/g, respectively, including the mesoporous surface area (166 m^2^/g) and mesoporous volume (0.16 cm^3^/g), respectively. Compared with the mBeta, the surface areas of Cu-mBeta, Bi-mBeta and Cu_x_Bi_y_-mBeta show obvious decrease after loading amount of copper and bismuth species, which is resulted from the generation of non-framework Cu or/and Bi ions (*i.e.*, CuO and Bi_2_O_3_) with the increase of Cu or Bi content, and inducing the collapse of pore structure of zeolite to some extent. Even so, the prepared Cu_1_Bi_1_-mBeta still keep high BET surface area and pore volume after doping with Cu and Bi species (Table 1), indicative of the unblocked mesoporous channels due to the high dispersity of Cu and Bi species. It is noted that the optimized Cu_1_Bi_1_-mBeta with the mesopore size of 3.6 nm shows high BET surface area (539 m^2^/g) and total pore volume (0.46 cm^3^/g).

**Figure 3:**
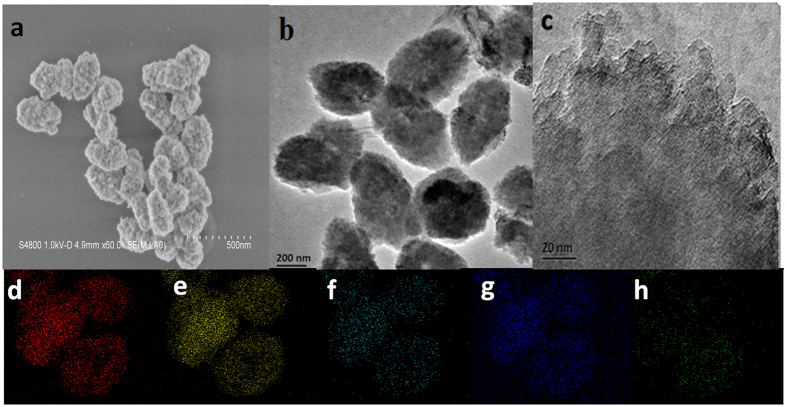
(**a**) Typical SEM images, (**b**) low-magnification TEM images, (**c**) high-magnification TEM images and (**d–h**) the corresponding element mappings of Si, O, Al, Cu and Bi of Cu_1_Bi_1_-mBeta.

The SEM image of as-prepared Cu_1_Bi_1_-mBeta, as shown in Fig. 3a, presents a rough surface morphology, demonstrating that the mesoporous structure has penetrated into the zeolite crystals by one-pot hydrothermal synthesis process. Additionally, no oxide aggregations can be found, as shown in Fig. 3a,b, indicating that the doped metal oxides are highly dispersed into the carrier mBeta. The clearly crystal lattices can be found in the high-magnification TEM image (Fig. 3c), confirming the zeolite crystallized structure. The element mapping in Fig. 3d–h further confirms that Cu and Bi species are highly dispersed into mesoporous zeolite Beta, which is consistent with the above results of XRD patterns.

### Spectroscopy Characteristics

**Figure 4:**
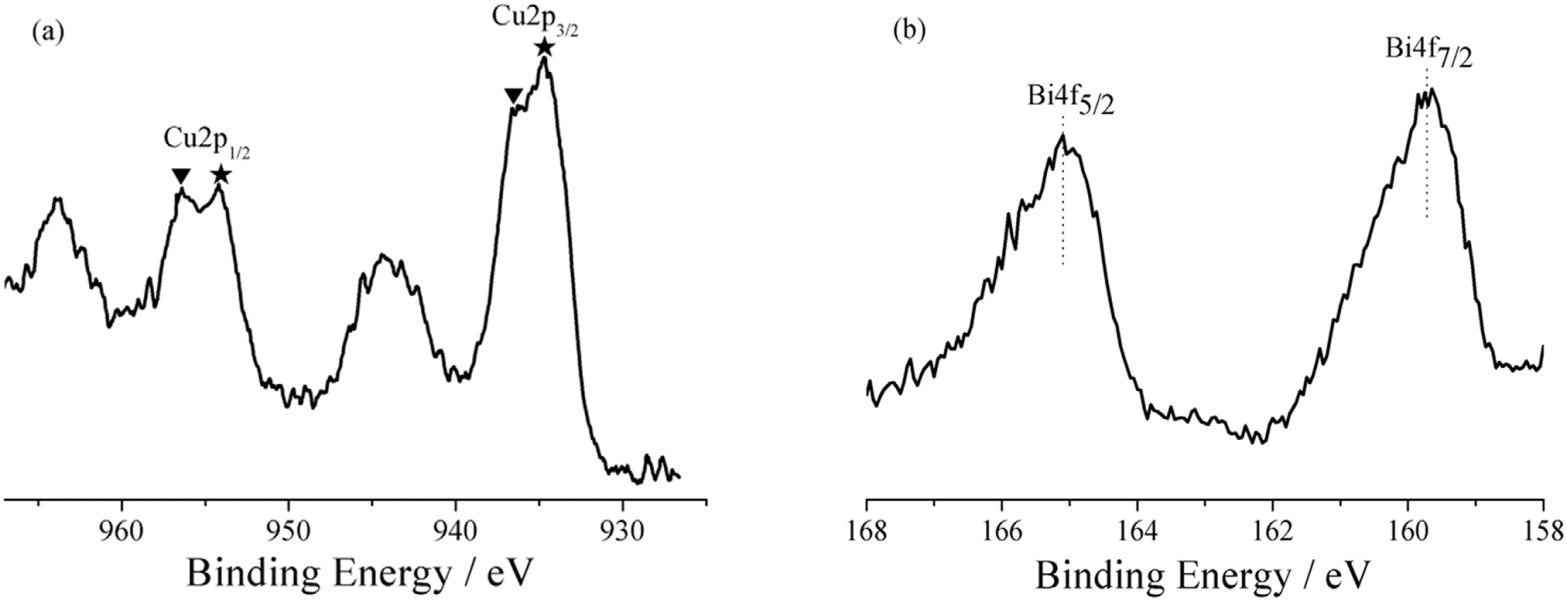
XPS spectra of (**a**) Cu 2p, (**b**) Bi 4f on the Cu_1_Bi_1_-mBeta catalyst.

The X-ray transmission spectroscopy (XPS) result of the Cu_1_Bi_1_-mBeta is show in Fig. 4a. The binding energy levels of Cu 2p_3/2_ and Cu 2p_1/2_ at around 934.5 eV and 954.2 eV, respectively (denoted with ★), accompanied by a shoulder peak of about 10 eV higher binding energy, are attributable to Cu(II). Additionally, the distinctive peaks at 936.6 eV and 956.4 eV, (denoted with ▼) can be ascribed to Cu(I), indicating that Cu species have variable valencies in the obtained sample Cu_1_Bi_1_-mBeta. In addition, the prepared Cu_1_Bi_1_-mBeta presents higher binding energy peak intensities of Cu(II) (934.5 eV) than that of Cu(I) (936.6 eV), as shown in Fig. 4a, suggesting that Cu_1_Bi_1_-mBeta contains much more amounts of Cu(II) than Cu(I)^15^,^16^. Figure 4b shows the Bi 4f XPS spectrum of Cu_1_Bi_1_-mBeta catalyst. Compared to pure Bi_2_O_3_ at 164.2 eV and 158.9 eV, the Cu_1_Bi_1_-mBeta shows the Bi 4f_5/2_ and Bi 4f_7/2_ a little higher binding energies at 164.6 eV and 159.5 eV, respectively, which is attributed to the interaction between Bi and Cu or the support mBeta^17^,^18^,^19^.

**Figure 5:**
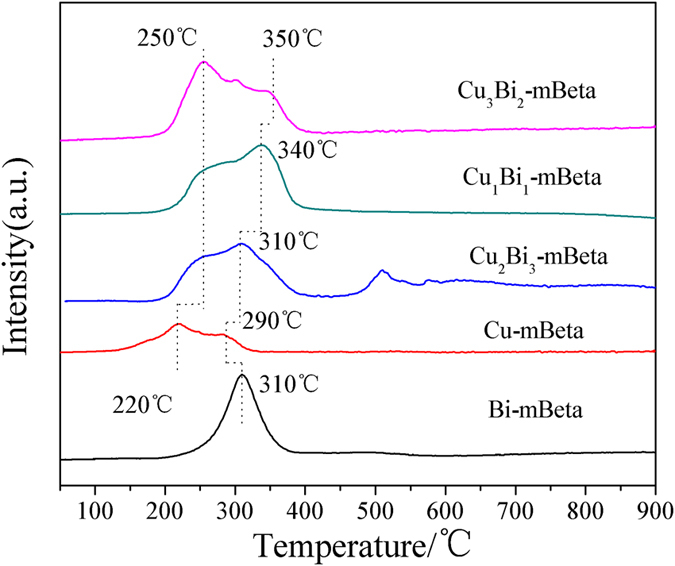
H_2_-TPR profiles of Cu-mBeta, Bi-mBeta and Cu_x_Bi_y_-mBeta samples.

The H_2_-TPR profiles of Cu-mBeta, Bi-mBeta and a series of Cu_x_Bi_y_-mbeta samples are shown in Fig. 5. It is found that the reference single loaded sample Cu-mBeta shows two weak reduction peaks at around 220 °C and 290 °C, indicating a two-step reduction of Cu^2+^ ions, *i.e.,* firstly to Cu^+^ and then to Cu^20^,^21^. In addition, the reduction peak at 310 °C of reference Bi-mBeta can be ascribed to the reduction of Bi_2_O_3_. The H_2_-TPR profiles of co-loaded samples Cu_x_Bi_y_-mBeta show a distinctively different redox behavior from either Cu-mBeta or Bi-mBeta, and present enhanced reduction peak at 250–350 °C, attributed to the strong interaction between the active species CuO and Bi_2_O_3_. In addition, the reduction peak gradually shifts toward lower temperature range (from 350 to 310 °C) with the increase of Bi content (Fig. 5 and Table 1), confirming that the addition of Bi_2_O_3_ is beneficial to promote the catalytic redox reaction^22^.

**Figure 6:**
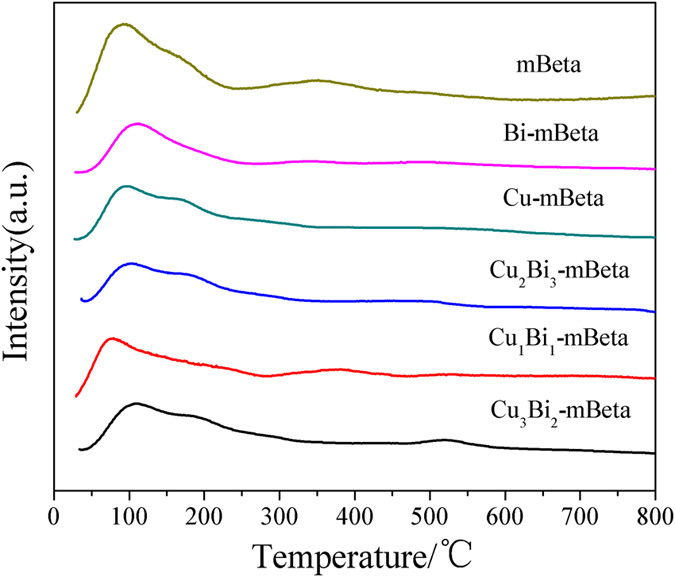
NH_3_-TPD profiles of mBeta, Cu-mBeta, Bi-mBeta and Cu_x_Bi_y_-mBeta samples.

**Figure 7:**
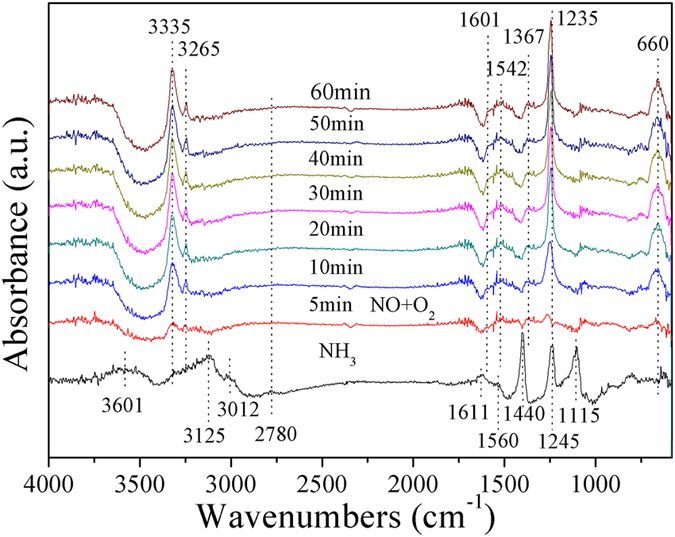
*In situ* DRIFTS spectra over Cu_1_Bi_1_-mBeta catalyst at 250 °C.

NH_3_-TPD experiments were carried out to obtain the acidity information of the prepared catalysts, as shown in Fig. 6. It is clear that a low-temperature peak at 150 °C and a high-temperature peak at 300 °C can be observed for the sample mBeta, which is assigned to weakly weak Lewis acid sites and strong Brønsted acid sites, respectively^23^,^24^. It is noted that all the samples Cu-mBeta, Bi-mBeta and Cu_x_Bi_y_-mBeta show weaker low-temperature peaks than that of the reference mBeta, owing to the part destruction of zeolite framework structure after introducing Cu and Bi species. However, it is interesting that only the Cu_1_Bi_1_-mBeta shows a similar desorption peak at high-temperature range (300–400 °C) to the mBeta, indicating that the Cu_1_Bi_1_-mBeta sample still keeps the strong acidity site of mBeta. The presence of rich acidic sites (Brønsted acid and Lewis acid) produced from the framework Al atoms and copper/bismuth species are helpful to the adsorption and activation of NH_3_, and thus producing many ammonia species, including NH_2_, coordinated NH_3_ and ionic NH_4_^+^, which can greatly promote the selective catalytic reduction of NO_x_, as shown in Fig. 7.

The reaction of adsorbed NH_3_ species towards NO + O_2_ was evaluated by the *in situ* IR spectra at 250 °C and the results are shown in Fig. 7. When the catalyst are exposed to NH_3_ for the 60 min and purged with N_2_, the peaks related to coordinated NH_3_ on Lewis acid sites (3125, 3002, 1611, 1245 and 1115 cm^−1^) and ionic NH_4_^+^ bound to Brønsted acid sites (3601 and 1440 cm^−1^) are clearly observed^15^,^25^,^26^,^27^. Afterwards, the coordinated NH_3_ on acidic sites could undergo the oxidative dehydrogenation to form NH_2_ species (1560 cm^−1^), then produce intermediate specie NH_2_NO when NO and O_2_ were added into reaction gas. It is noted that all the ammonia species, including NH_2_, coordinated NH_3_ and ionic NH_4_^+^ bound, disappeared after NO + O_2_ purge, indicating that those ammonia species could participate in the reduction of NO_x_. Meanwhile, when NO and O_2_ were added into reaction gas, the bands at 1235, 1367, 1542 and 1601 cm^−1^ could be detected in IR spectra. Thereinto, the bands at 1235 and 1367 cm^−1^ were assigned to monodentate nitrate, while the bands at 1542 and 1601 cm^−1^ are associated with bidentate nitrate and adsorbed NO_2_, respectively^15^,^25^. More interestingly, two peculiar peaks at 3335 and 3265 cm^−1^ related to the adsorbed NH_3_ on acid sites became stronger with the increase of exposing time in the NO + O_2_, indicating that some acidic sites on the surface of the catalyst were released and then preferably adsorbed the NH_3_ after NO and O_2_ pass over the catalyst.

### Catalytic performance

**Figure 8:**
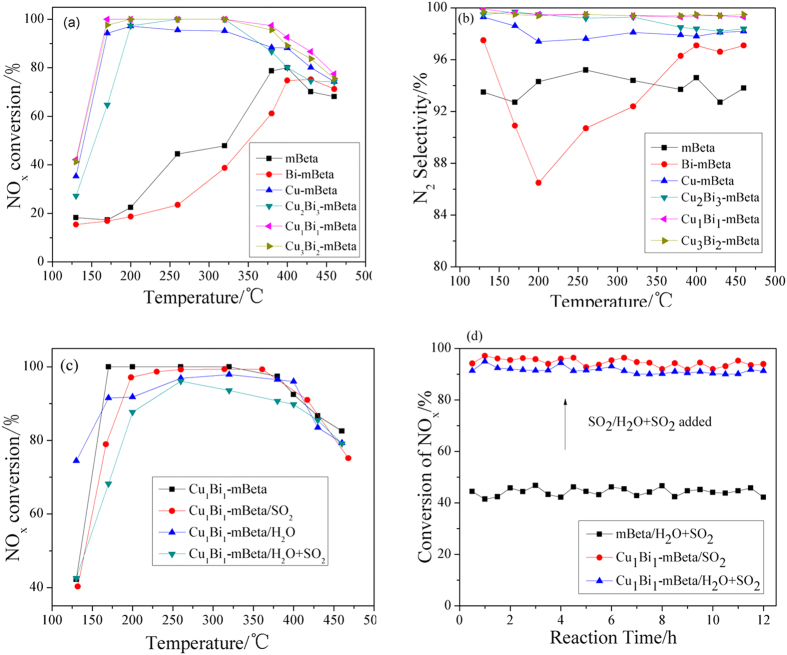
NO_x_ conversion (**a**), N_2_ selectivity (**b**), H_2_O and SO_2_ resistance (**c**) and durability test at 250°C (**d**) for the NH_3_-SCR over mBeta, Cu-mBeta, Bi-mBeta and a series of Cu_x_Bi_y_-mBeta catalysts with different Cu/Bi ratios (500 ppm NO, 500 ppm NH_3_, 0 or 3% H_2_O, 0 or 50 ppm SO_2_, 5% O_2_, balance Ar, GHSV = 64 000 h^−1^).

Figure 8 shows the NH_3_-SCR results of mBeta, Cu-mBeta, Bi-mBeta and a series of Cu_x_Bi_y_-mBeta catalysts. The NO_x_ conversions over the prepared catalysts under high hourly space velocity of 64000 h^−1^ are shown in Fig. 8a. Compared with the references mBeta, Bi-mBeta and Cu-mBeta, the sample Cu_x_Bi_y_-mBeta show higher catalytic activity for the SCR of NO_x_. Especially, the optimized sample Cu_1_Bi_1_-mBeta with the 4.38 wt% Cu and 4.83 wt% Bi exhibits the highest catalytic performance, *i.e.*, the NO_x_ conversion efficient is above 90% within the wide operation temperature window of 170 °C to 400 °C. While, the excess doping of Cu and Bi species could induce the formation of oxides aggregates and thus decrease the active surface area (Table 1), *e.g.,* the excess Bi would cover part of active center and results a lower catalytic activity. Therefore, the optimized sample Cu_1_Bi_1_-mBeta with the 1:1 ratio of Cu and Bi shows the highest catalytic performance. The results of N_2_ selectivity of these catalysts were shown in Fig. 8b. For comparison, the reference Bi-mBeta shows a low N_2_ selectivity, particular in 200 °C, owing to the part destruction of zeolite framwork with the addition of Bi species, as demonstrated by XRD results, which can affect the strong acidic sites (Fig. 6), thus decrease the adsoption ability of NH_3_ on the reference Bi-mBeta. Furthermore, the Cu_1_Bi_1_-mBeta catalyst also presents as high as up to 100% N_2_ selectivity in the whole temperature range investigated. It is believed that the high dispersity of active species Cu and Bi on the support mesoporous zeolite, the richer acidic sites and the strong interaction between the active copper and bismuth species on the Cu_1_Bi_1_-mBeta could be main contributions to the catalytic activity.

It is well known that the real diesel exhaust presents large number of water vapor and trace S compounds, therefore, the effects of H_2_O and SO_2_ on the SCR catalytic activity are also investigated on the samples Cu_1_Bi_1_-mBeta. It is evident that compared with the reference mBeta, Cu_1_Bi_1_-mBeta catalyst shows an excellent SO_2_ resistance in the NH_3_-SCR above 200 °C (Fig. 8c). Even in the co-presence of H_2_O and SO_2_, the Cu_1_Bi_1_-mBeta catalyst still exhibits high activity of over 85% NO_x_ conversion from 200 °C to 430 °C. The results of durability tested at 250 °C, as shown in Fig. 8d, indicate that the Cu_1_Bi_1_-mBeta is very stable in the presence of SO_2_ (or H_2_O and SO_2_). It is believed that the highly crystalline zeolite framework enables the catalyst to keep good stability against H_2_O, and the highly dispersity active speicies Bi can improve the SO_2_ resistance to some extent^13^, which is very important for the NH_3_-SCR of NO_x_ in the co-existence of H_2_O and SO_2_.

## Discussion

**Figure 9:**
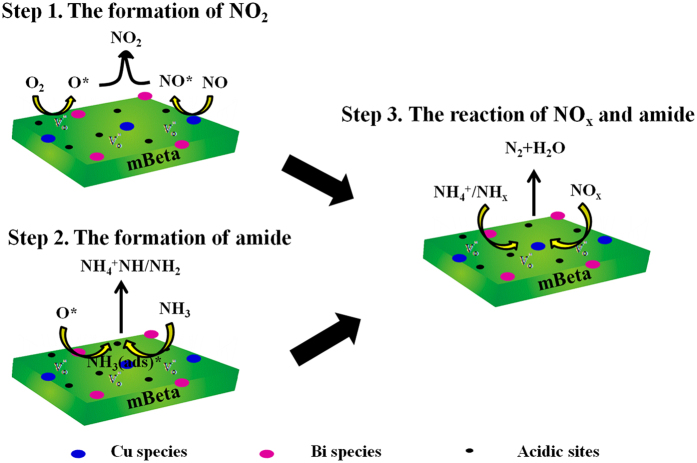
A possible reaction mechanism of NH_3_-SCR of NO on Cu_1_Bi_1_-mBeta catalyst.

Based on the above results and discussions, a possible catalytic reaction mechanism for the SCR of NO_x_ was proposed, as illustrated in Fig. 9. Firstly, there are a large amount of oxygen vacancies (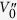) presented in the sample support mBeta due to the doping of hetero atoms Cu^n+^, Bi^3+^ and Al^3+^ in the [SiO_4_], leading to the generation of numerous surface activated oxygen (O^*^) by adsorbing the O_2_, as shown in Step 1 (Fig. 9). Meanwhile, the existence of Bi_2_O_3_ was reported^22^,^28^ to be beneficial for the reduction of Cu^2+^ to Cu^+^, and NO could be easily adsorbed and activated by the Cu^n+^ and generated the 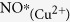 and 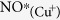 at higher temperatures or lower temperatures, respectively^29^,^30^. Afterwards, these activated 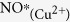 and 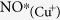 could react with the surface activated oxygen (O^*^) and thus produce large numbers of NO_2_, as shown in Step 1 (Fig. 9). When the concentration ratio of NO and NO_2_ in reaction gas reaches to 1:1, the quick SCR reaction (NO + NO_2_ + 2NH_3_ = 2N_2 _+ 3H_2_O) occurs, during which the NO_x_ conversion efficiency at low temperature can be greatly improved. Secondly, the presence of rich acidic sites (Brønsted acid and Lewis acid) produced from the framework Al atoms and copper/bismuth species is helpful to the adsorption and activation of NH_3_, *i.e.*, the NH_3_ adsorbed on strong Brønsted acid sites could be activated and generate NH_4_^+^, which was discovered from the *in situ* DRIFTs (Fig. 7), as shown in Step 2 (Fig. 9). Meanwhile, the NH_3_ molecules could also be adsorbed on the weak Lewis acid sites to generate NH_3(ads)_ and react with the activated oxygen (O^*^) to produce the amines NH/NH_2_, as confirmed by the *in situ* DRIFTs (Fig. 7). Finally, the produced amide NH/NH_2_ and NH_4_^+^ could directly react with NO_x_ on the highly dispersed active sites and generate N_2_, as shown in Step 3 (Fig. 9). It is believed that the existence of highly dispersed varied valence Cu species and acidic sites can accelerate the adsorption and activation of NO and NH_3_ in this reaction system, which greatly increases the NH_3_-SCR in the removal of NO_x_.

In conclusion, a series of Cu_x_Bi_y_-mBeta catalysts have been prepared by a facile one-pot hydrothermal treatment approach. The optimized Cu_1_Bi_1_-mBeta shows an excellent NH_3_-SCR activity and high N_2_ selectivity (closely to 100%) toward NO_x_ in a broad operation temperature window (170–400 °C). On the one hand, the mesopores structure is helpful for the homogeneous dispersion of active species, which can improve the diffusion and transport of reactants and products. Also, the highly crystalline zeolite framework enables the catalyst to keep good resistence toward water vapor. On the other hand, the highly dispersity of copper and bismuth active species, as well as the synergetic catalytic effect between copper and bismuth species and the richer acidic sites of the zeolite promote the reduction of CuO and generation of intermediate NO_2_. Meanwhile, the large number of amide NH/NH_2_ and NH_4_^+^ generated from NH_3_ adsorption, as the key intermediates, greatly accelerate the selective catalytic reduction of NO_x_. More interesting, the prepared catalyst shows good durability and high resistance against H_2_O and SO_2_, which could also be ascribed to the crystalline zeolite framework and the highly dispersity active sites. The CuBi-mBeta catalyst with distinctive mirco-mesoporous structure demonstrates excellent NH_3_-SCR activity and high stability, which, as we believe, will present promising prospect in the practical application of catalytic purification of diesel exhausts.

## Methods

### Preparation of the mesoporous zeolite Beta (mBeta)

Typically, 0.05 g NaCl and 0.15 g KCl were added into 2 mL distilled water and 14.4 g TEAOH solution. Afterwards, 3.9 g H_2_SiO_3_ was dissolved into the above mentioned solution and stirred at 313 K for 6 h. Next, the solution containing 0.033 g NaOH, 0.17 g NaAlO_2_ and 2 mL distilled water was slowly added into the resultant solution and further stirred at 313 K for 6 h. Finally, 0.5 g CTAB solution was added into the obtained solution and further stirred at 353 K for 8 h. The obtained mixed solution was hydrothermally treated for 48 h at 423 K. Subsequently, the products were washed with distilled water and dried at 383 K for 12 h. The final product mBeta was obtained after calcinations at 823 K for 6 h to remove any organics.

### Preparation of Cu_1_Bi_1_-mBeta

Typically, 0.05 g NaCl and 0.15 g KCl were added into 2 mL distilled water and 14.4 g TEAOH solution. Afterwards, 3.9 g H_2_SiO_3_ was dissolved into the above mentioned solution and stirred at 313 K for 6 h. Then, the solution containing 0.033 g NaOH, 0.17 g NaAlO_2_ and 2 mL distilled water was slowly added into the resultant solution and stirring. Next, 1 mmol Cu(NO_3_)_2_•3H_2_O, 1 mmol Bi(NO_3_)_3_•5H_2_O and 2 mL distilled water was slowly added into the above precursor solution and further stirred at 313 K for 6 h. Then, 0.5 g CTAB solution was added into the obtained solution and further stirred at 353 K for 8 h. Next, the obtained mixed solution was hydrothermally treated for 48 h at 423 K. Subsequently, the products were washed with distilled water and dried at 383 K for 12 h. The final product, Cu_1_Bi_1_-mBeta, was obtained after calcinations at 823 K for 6 h to remove any organics.

For comparison, the Cu-mBeta, Bi-mBeta and Cu_x_Bi_y_-mBeta were synthesized by a facile one-pot hydrothermal treatment approach, similarly to the above process of Cu_1_Bi_1_-mBeta. Herein, the x and y represent the millimole amount of Cu and Bi in the initial precursor solution, respectively. In addition, the final pH value of the synthetic gel including copper and bismuth species is about 11.

### Sample characterization

The XRD patterns were recorded on a Rigaku D/Max-2200PC X-ray diffractometer using Cu target at 40 kV and 40 mA. The N_2_ adsorption and desorption measurements were performed using Micromeritics Tristar 3000 at 77 K. The total surface area and pore volume were calculated using the BET and BJH method. Field emission scanning electron microscopy (SEM) analysis was performed on a JEOL JSM6700F electron microscope. Field emission transmission electron microscopy (TEM) analysis was conducted with a JEOL 200CX electron microscope operated at 200 keV. X-ray photoelectron spectroscopy (XPS) signals were collected on a Thermo Scientific ESCALAB 250 instrument using monochromated Al X-ray resource at 1486.6 eV operated at 15 kW. The temperature-programed reduction with hydrogen (H_2_-TPR) and temperature programmed desorption of ammonia (NH_3_-TPD) were performed on Micromeritics Chemisorb 2750 instrument attached with ChemiSoft TPx software. TPR was carried out from room temperature to 900 °C under 5% H_2_ in Ar at a flow rate of 25 mL/min. The H_2_ signal was detected by a thermal conductivity detector (TCD). The NH_3_-TPD of the samples was carried out from room temperature to 800 °C at a flow rate of 25 mL/min. The amount of NH_3_ desorbed was measured using a thermal conductivity detector (TCD). *In situ* diffuse reflectance infrared Fourier transform spectroscopy (DRIFTS) was performd on a Bruker spectrometer equipped with an MCT detector. Prior to each experiment, the sample was pretreated at 400 °C for 1 h in a flow of N_2_ and then cooled down to 200 °C. The background spectrum was collected in flowing N_2_ and automatically subtracted from the sample spectrum. The reaction conditions were controlled as follows: 100 mL min^−1^ total flow rate, 500 ppm NH_3_, 500 ppm NO, 5% O_2_ and N_2_ as the balance. All spectra were recorded by accumulating 100 scans with a resolution of 4 cm^−1^. The contents of Cu and Bi were measured by using Inductively Coupled Plasma Atomic Emission Spectroscopy (ICP-AES) analyzer on a Vista AX.

### Catalytic activity test

The catalysis measurements were carried out in a fixed-bed quartz reactor using 0.2 g catalyst of 40–60 meshes. Before catalytic test, the catalysts were dried at 423 K for 16 h. The feed gas mixture contained 500 ppm NO, 500 ppm NH_3_, 0 or 3% H_2_O, 0 or 50 ppm SO_2_, 5% O_2_ and Ar as the balance gas. The total flow rate of the feed gas was 300 mL/min, corresponding to a GHSV of 64,000 h^−1^. The composition of the product gas was analyzed by a chemiluminescence NO/NO_2_ analyzer (Thermal Scientific, model 42i-HL) and gas chromatograph (Shimadzu GC 2014 equipped with Porapak Q and Molecular sieve 5A columns). The activity data were collected when the catalytic reaction practically reached steady-state condition at each temperature.

The NO_x_ (*X*_NOx_) and NH_3_ (*X*_NH3_) conversions and N_2_ selectivity (*S*_N2_) were calculated as


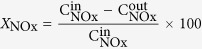



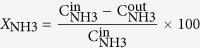



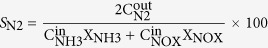


where NO_x_ includes NO and NO_2_, *C*_*i*_ presents the concentration of the “i” species, and the “in” and “out” present the gas concentration of inlet and the exit of the reactor, respectively.

## Additional Information

**How to cite this article**: Xie, Z. *et al*. One-pot hydrothermal synthesis of CuBi co-doped mesoporous zeolite Beta for the removal of NO_x_ by selective catalytic reduction with ammonia. *Sci. Rep.* **6**, 30132; doi: 10.1038/srep30132 (2016).

## Figures and Tables

**Table 1:** The composition and structural parameters of the synthesized catalysts.

Sample	Cu^a^ (wt%)	Bi^a^ (wt%)	S_total_ (m^2^/g)	S_meso_^b^ (m^2^/g)	V_total_ (cm^3^/g)	V_meso_^c^ (cm^3^/g)	Pore diameter (nm)
mBeta	—	—	556	166	0.34	0.16	3.8
Bi-mBeta	—	4.78	413	193	0.31	0.21	2.9
Cu-mBeta	4.4-	—	458	321	0.33	0.27	2.8
Cu_2_Bi_3_-mBeta	4.32	7.23	415	154	0.36	0.24	3.9
Cu_1_Bi_1_-mBeta	4.38	4.83	539	304	0.46	0.36	3.6
Cu_3_Bi_2_-mBeta	6.58	4.77	523	270	0.48	0.35	3.7

^a^Measured by ICP-AES.

^b^S_meso_ was given by the difference between S_total_ and S_micro_.

^c^V_meso_ was given by the difference between V_total_ and V_micro_.

## References

[b1] Su W. K., Chang H. Z., Peng Y., Zhang C. Z. & Li J. H. Reaction pathway investigation on the selective catalytic reduction of NO with NH_3_ over Cu/SSZ-13 at low temperatures. Environ. Sic. Technol. 49, 467–473 (2015). 10.1021/es503430w 25485842

[b2] Zhang Q. L. . Low-temperature selective catalytic reduction of NO with NH_3_ over monolith catalyst of MnO_x_/CeO_2_-ZrO_2_-Al_2_O_3_. Catal. Today 175, 171–176 (2011).

[b3] Peng Y., Qu R. Y., Zhang X. Y. & Li J. H. The Relationship between Structure and Activity of MoO_3_-CeO_2_ Catalysts for NO Removal: Influences of Acidity and Reducibility. Chem. Commun. 49, 6215–6217 (2013). 10.1039/c3cc42693a 23736146

[b4] Jin R. B. . The role of cerium in the improved SO_2_ tolerance for NO reduction with NH_3_ over Mn-Ce/TiO_2_ catalyst at low temperature. Appl. Catal. B 148–149, 582–588 (2014).

[b5] Chang X. F., Lu G. Z., Guo Y., Wang Y. Q. & Guo Y. L. A high effective adsorbent of NO_x_: Preparation, characterization and performance of Ca-beta zeolites. Microporous Mesoporous Mater. 165, 113–120 (2013).

[b6] Lethbridge Z. A. D., Williams J. J., Walton R. I., Evans K. E. & Smith C. W. Methods for the synthesis of large crystals of silicate zeolites. Microporous Mesoporous Mater. 79, 339–352 (2005).

[b7] Xu L. . Enhancement of low-temperature activity over Cu-exchanged zeolite beta from organotemplate-free synthesis for the selective catalytic reduction of NO_x_ with NH_3_ in exhaust gas streams. Microporous Mesoporous Mater. 200, 304–310 (2014).

[b8] Zhu J. . Highly Mesoporous Single-Crystalline Zeolite Beta Synthesized Using a Nonsurfactant Cationic Polymer as a Dual-Function Template. J. Am. Chem. Soc. 136, 2503–2510 (2014). 24450997 10.1021/ja411117y

[b9] Chen C. Y. . Enhanced performance in catalytic combustion of toluene over mesoporous Beta zeolite-supported platinum catalyst. Appl. Catal. B 140–141, 199–205 (2013).

[b10] Yin C. Y. . One-step synthesis of hierarchical mesoporous zeolite Beta microspheres from assembly of nanocrystals. J. Colloid. Interface Sci. 397, 108–113 (2013). 23481514 10.1016/j.jcis.2013.02.006

[b11] Baerdemaeker T. D. . Catalytic applications of OSDA-free Beta zeolite. J. Catal. 308, 73–81 (2013).

[b12] Deka U., Lezcano-Gonzalez I., Weckhuysen B. M. & Beale A. M. Local Environment and Nature of Cu Active Sites in Zeolite-Based Catalysts for the Selective Catalytic Reduction of NO_x_. ACS Catal. 3, 413–427 (2013).

[b13] Karlsson H. T. & Rosenberg H. S. Flue gas denitrification. Selective catalytic oxidation of NO to NO_2_. Ind. Eng. Chem. Proc. Des. Dev. 23, 808–814 (1984).

[b14] Zhou X. X. . A facile one-pot synthesis of hierarchically porous Cu(I)-ZSM-5 for radicals-involved oxidation of cyclohexane. Appl. Catal. A 451, 112–119 (2013).

[b15] Zhang R. R., Li Y. H. & Zhen T. L. Ammonia selective catalytic reduction of NO over Fe/Cu-SSZ-13. RSC Adv. 4, 52130–52139 (2014).

[b16] Chadwick D. & Hashemi T. Adsorbed corrosion inhibitors studied by electron spectroscopy: Benzotriazole on copper and copper alloys. Corros. Sci 18, 39–51 (1978).

[b17] Bian Z. F. . Self-Assembly of Active Bi_2_O_3_/TiO_2_ Visible Photocatalyst with Ordered Mesoporous Structure and Highly Crystallized Anatase. J. Phys. Chem. C 112, 6258–6262 (2008).

[b18] Shan W. J., Hu Y., Zheng M. M. & Wei C. H. The enhanced photocatalytic activity and self-cleaning properties of mesoporous SiO_2_ coated Cu-Bi_2_O_3_ thin films. Dalton Trans. 44, 7428–7436 (2015). 25801807 10.1039/c5dt00381d

[b19] Jiang H.-Y. . Efficient organic degradation under visible light by α-Bi_2_O_3_ with a CuO_x_-assistant electron transfer process. Appl. Catal. B 163, 267–276 (2015).

[b20] Richter M. . Gas-phase carbonylation of methanol to dimethyl carbonate on chloride-free Cu-precipitated zeolite Y at normal pressure. J. Catal. 245, 11–24 (2007).

[b21] Xue J. J. . Characterization of copper species over Cu/SAPO-34 in selective catalytic reduction of NO_x_ with ammonia: Relationships between active Cu sites and de-NO_x_ performance at low temperature. J. Catal. 297, 56–64 (2013).

[b22] Yang G. H. . MCM-41 supported CuO/Bi_2_O_3_ nanoparticles as potential catalyst for 1,4-butynediol synthesis. Ceram. Int. 40, 3969–3973 (2014).

[b23] Zhou X. X. . Dual-mesoporous ZSM-5 zeolite with highly b-axis-oriented large mesopore channels for the production of benzoin ethyl ether. Chem.-Eur. J. 19, 10017–10023 (2013). 23775816 10.1002/chem.201300245

[b24] Wang D. . A comparison of hydrothermal aging effects on NH_3_-SCR of NO_x_ over Cu-SSZ-13 and Cu-SAPO-34 catalysts. Appl. Catal. B 165, 438–445 (2015).

[b25] Liu Z. M. . A superior catalyst with dual redox cycles for the selective reduction of NO_x_ by ammonia. Chem. Commun. 49, 7726–7728 (2013). 10.1039/c3cc43041c 23877875

[b26] Meng D. M. . A Highly Effective Catalyst of Sm-MnO_x_ for the NH_3_-SCR of NO_x_ at Low Temperature: Promotional Role of Sm and Its Catalytic Performance. ACS Catal. 5, 5973–5983 (2015).

[b27] Yu T. . Recent NH_3_-SCR Mechanism Research over Cu/SAPO-34 Catalyst. J. Phys. Chem. C 118, 6565–6575 (2014).

[b28] Lin G. . Universal Preparation of Novel Metal and Semiconductor Nanoparticle-Glass Composites with Excellent Nonlinear Optical Properties. J. Phys. Chem. C 115, 24598–24604 (2011).

[b29] Chen B. H., Xu R. N., Zhang R. D. & Liu N. Economical Way to Synthesize SSZ-13 with Abundant Ion-Exchanged Cu^+^ for an Extraordinary Performance in Selective Catalytic Reduction (SCR) of NO_x_ by Ammonia. Environ. Sci. Technol. 48, 13909–13916 (2014). 25365767 10.1021/es503707c

[b30] Zhang R. Q. . NO Chemisorption on Cu/SSZ-13: A Comparative Study from Infrared Spectroscopy and DFT Calculations. ACS Catal. 4, 4093–4105 (2014).

